# Irreversible Electroporation: Background, Theory, and Review of Recent Developments in Clinical Oncology

**DOI:** 10.1089/bioe.2019.0029

**Published:** 2019-12-12

**Authors:** Kenneth N. Aycock, Rafael V. Davalos

**Affiliations:** Department of Biomedical Engineering and Mechanics, Virginia Tech-Wake Forest University, Blacksburg, Virginia.

**Keywords:** irreversible electroporation, pulsed electric field, electroporation, interventional oncology

## Abstract

Irreversible electroporation (IRE) has established a clinical niche as an alternative to thermal ablation for the eradication of unresectable tumors, particularly those near critical vascular structures. IRE has been used in over 50 independent clinical trials and has shown clinical success when used as a standalone treatment and as a single component within combinatorial treatment paradigms. Recently, many studies evaluating IRE in larger patient cohorts and alongside other novel therapies have been reported. Here, we present the basic principles of reversible electroporation and IRE followed by a review of preclinical and clinical data with a focus on tumors in three organ systems in which IRE has shown great promise: the prostate, pancreas, and liver. Finally, we discuss alternative and future developments, which will likely further advance the use of IRE in the clinic.

## Introduction

Electroporation is a biophysical phenomenon in which cellular membranes exhibit increased permeability to ions and macromolecules when exposed to external electric fields. Although the exact mechanisms of electroporation have not been fully elucidated, the scientific community has mostly come to agreement that permeabilizing nanoscale defects or “nanopores” are formed in cellular membranes upon exposure to high-amplitude electric fields of sufficient duration.^1^ This phenomenon is manifested in two distinct forms: reversible electroporation, in which permeabilizing structures are transient and membrane integrity is quickly recovered; and irreversible electroporation (IRE), in which permeabilization disrupts cellular homeostasis and leads to cell death.

### The discovery of reversible electroporation

The earliest reports of electroporation date back over two centuries, but the most widely recognized initial discoveries originate in the 1950s when Stampfli and Willi studied the “electrical breakdown” of nodes of Ranvier extracted from frogs.^2,3^ A decade later, intense electric fields were used to nonthermally kill microorganisms and to induce changes in permeability of vesicular membranes, leading to the release of catecholamines.^4,5^ A series of investigations by Kinosita and Tsong in the 1970s showed that high-amplitude pulsed electric fields (PEFs) could be tuned to generate pores of different sizes in erythrocyte membranes, allowing for selective internalization of normally impermeant molecules.^6,7^ They also demonstrated the increase in conductivity that follows the application of high voltage pulses and the ability to directly cause hemolysis with induced electric fields.^8,9^ These early contributions provided evidence supporting the theory of aqueous pore formation, which was first published in 1979.^10^ We acknowledge the perpetuity of this work as the fundamental principles introduced still underlie our current understanding of electroporation.

Within the next decade, seminal investigations established the ability of PEFs to increase uptake of genes and chemotherapeutics, leading to the development of the prominent fields now known as electrochemotherapy (ECT) and electrogene transfer (EGT).^11–13^ As its name implies, ECT utilizes reversible electroporation to locally increase cellular uptake of chemotherapeutics, namely bleomycin or cisplatin, which allows for lower drug concentrations and can limit off-target toxicity. EGT employs similar techniques to introduce plasmid DNA into cells. This technique is now widely used for laboratory transfection assays, but its clinical utility is still being developed.

The aforementioned studies, along with many others, laid the framework for clinical applications of electroporation, which focused on enhancing transmembrane transport of existing biological and pharmaceutical agents until the 21st century. During these two decades, electroporation was not used directly to injure cells, and IRE was largely viewed as an undesirable side effect of overtreatment.

### IRE as a tissue ablation modality

In 2005, however, Davalos et al. proposed IRE as a standalone soft tissue ablation technique.^14^ Their original work showed numerically that electric fields capable of nonthermal IRE exist and could destroy clinically relevant volumes of tissue. Unlike existing focal ablation techniques that indiscriminately eliminate all biomolecules within a certain proximity, IRE allows for preservation of collagenous and other protein and/or lipid-based structures including vasculature^15^ and ductal networks.^16^ It has also been shown that carefully planned IRE treatments cause minimal long-term damage to myelinated neurons, but that overtreatment or direct physical penetration can injure these structures.^17^ The inherent advantages of IRE compared with other focal cancer therapies, such as reduced treatment time, reduced vascular complications, decreased risk of overtreatment, and minimized heat-sink effect when administered near vessels, led to an array of early publications evaluating the efficacy and feasibility of IRE to nonthermally ablate healthy and malignant soft tissues including the liver,^18–20^ prostate,^21,22^ pancreas,^23^ kidney,^24^ lung,^25,26^ and brain.^27–30^ Hundreds of follow-up studies have scrutinized the application of IRE in these tissues and others. Importantly, a large number of preclinical and clinical reports have been published in recent years. This review will serve to present these results in an organized manner to serve as a comprehensive reference for the interested reader. To maintain brevity, our discussion will focus on treatments in the prostate, pancreas, and liver, as tumors in these tissues present obvious difficulties that IRE has the potential to address.

## Mechanisms of Electroporation and IRE

### A brief overview of pore formation theory

Although fundamentals of pore formation are not the focus of this review, we provide a brief summary. A number of exceptional publications^31–35^ have examined the mechanistic events leading to permeabilization in much more detail than offered here.

**FIG. 1.**
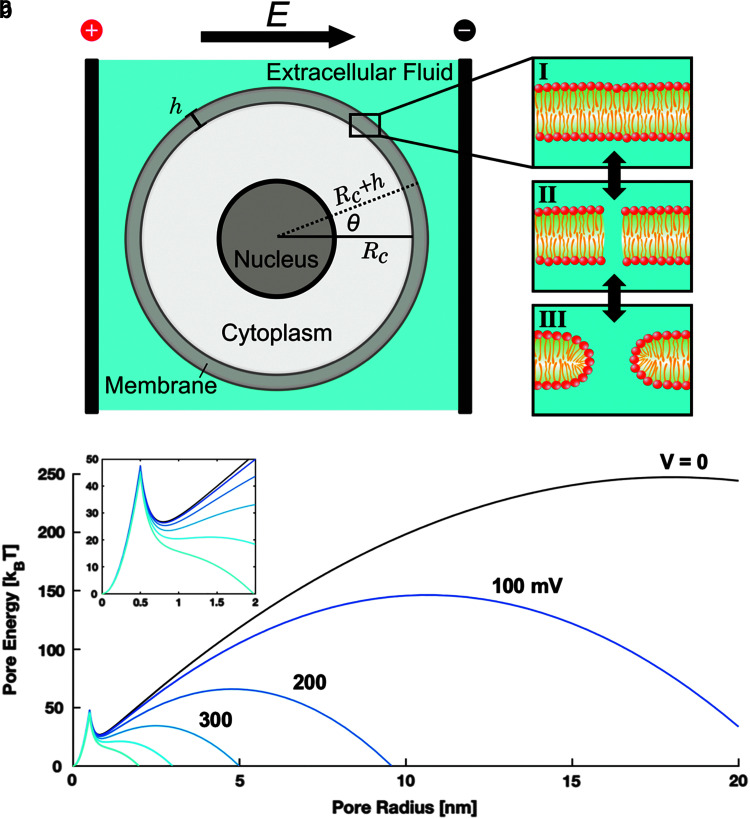
Pore formation is a stochastic process manifested in the lipid bilayer. The behavior of a cell exposed to an external electrical stimulus **(a)** depends on the amplitude and temporal characteristics of the field. Under physiological conditions **(I)**, the lipid bilayer is a stable barrier exhibiting permeability only to select molecules. After exposure to an intense electric field, hydrophobic pores appear immediately **(II)** and stabilize after reorientation of lipid head groups **(III)**, allowing for passage of previously impermeable molecules. The asymptotic model proposed by Neu and Krassowska^45^ shows that **(b)** the free energy of induced pores decreases with increasing transmembrane potential beyond a critical radius.

Biological membranes are organized into lipid bilayers (Fig. 1a). In aqueous solution, the amphiphilic fatty acids spontaneously form a membrane composed of two identical leaflets with opposite orientations since this is the most energetically favorable configuration. The outer face of either leaflet is composed of hydrophilic head groups that interact with the aqueous solution, whereas the innermost core is formed of hydrophobic tails. Physiologically, the lipid bilayer serves as a semipermeable barrier that separates the cytosol from extracellular fluid, only allowing diffusion of certain small uncharged and/or hydrophobic molecules. Large or charged molecules are transported through discrete transmembrane proteins organized into channels or pumps. Although the cell membrane is structurally stable, the fatty acids are held together by weak van der Waals forces, creating a “fluid-like” structure in which each fatty acid is constantly moving within the bilayer, mostly laterally within the same leaflet. This property gives the membrane the ability to allow passage of small molecules, to externalize proteins and waste, and to internalize molecules critical to intracellular processes.

The unique properties of the cell membrane allow aqueous pores to spontaneously form due to strong interactions between water molecules on either side, especially under environmental influences such as thermal fluctuations^36,37^ or mechanical insult.^38^ Although these nanopores can appear spontaneously, external electric fields (Fig. 1a) lower the activation energy necessary for the stochastic pore formation process, resulting in the production of pores at a higher rate.^31^

When the electrical stimulus is of sufficient strength, water dipoles on either side of the bilayer reorient to the field and their interaction becomes favorable.^39^ Initially, the water column spanning the membrane is highly unstable, forming hydrophobic pores or “water wires” (Fig. 1a-II).^40^ As electrical energy is delivered to the system and water molecules penetrate the membrane, many of the initial structures evolve into long-lived hydrophilic pores.^41^ This transition is mediated by reorientation of the polar fatty acid head groups into a more energetically favorable alignment, thereby stabilizing the pore.^10,35,40^ Simulations predict that hydrophobic pores are <1 nm in diameter and reseal within milliseconds, whereas hydrophilic pores are roughly 1–10 nm in diameter and reseal within minutes to hours.^33,35,41^

### Mathematical determinants of pore formation

Under physiological conditions, a biological cell will maintain an endogenous transmembrane potential (TMP), or resting potential, in which the inside of the cell has a slightly negative charge (−50 to −90 mV) relative to its environment. Under an applied field, an induced TMP, ΔΨ_*m*_, also arises across the membrane. Schwan was among the first to develop a mathematical description for ΔΨ_*m*_.^42^ Now bearing his name, this intuitive analytical equation assumes that the cell is isolated and has a spherical geometry with radius *R_c_* and membrane thickness *h* (Fig. 1a). The steady-state equation is relevant for low-frequency pulses (pulse widths >>1 μs) after the induced TMP has reached its maximum:

(1) $$\Delta \Psi_{m}\left(r,\theta \right)=\Delta \Psi_{m}\left(R_{c},\theta \right)-\Delta \Psi_{m}\left(R_{c}+h,\theta \right)=f_{s}ER_{c}cos\theta ,$$

where *E* is the magnitude of the homogenous applied field, *θ* is the polar angle between the normal vector of the applied field and the site on the membrane at which ΔΨ_*m*_ is evaluated, and *f_s_* is a dimensionless term relating the electrical properties of each component:

(2) $$f_{s}=\frac{3\sigma_{e}\left[3hR_{c}^{2}\sigma_{i}+\left(3h^{2}R_{c}-h^{3}\right)\left(\sigma_{m}-\sigma_{i}\right)\right]}{2R_{c}^{3}\left(\sigma_{m}+2\sigma_{e}\right)\left(\sigma_{m}+\frac{1}{2}\sigma_{i}\right)-2{\left(R_{c}-h\right)}^{3}\left(\sigma_{e}-\sigma_{m}\right)\left(\sigma_{i}-\sigma_{m}\right)}$$

In this equation, σ_*i*_, σ_*m*_, σ_*e*_ and represent the conductivity of the cytosol, membrane, and extracellular fluid, respectively. To evaluate the transient behavior of the induced TMP, dielectric permittivity of the lipid bilayer (ε_*m*_) must be considered, and the equation is written as:

(3) $$\Delta \Psi_{m}\left(r,\theta ,t\right)=f_{s}ER_{c}cos\theta \left(1-e^{{-}^{t/\tau_{m}}}\right).$$

This equation assumes that the intracellular and extracellular fluid permittivity is negligible. It is valid for sinusoidal fields with frequencies below 1 MHz and rectangular pulses longer than 1 μs.^43^ In Equation (3), τ_*m*_ is the membrane charging constant given by:

(4) $$\tau_{m}=\frac{R_{c}\varepsilon_{m}}{2h\left(\frac{\sigma_{i}\sigma_{e}}{\sigma_{i}+2\sigma_{e}}\right)+R_{c}\sigma_{m}}.$$

In many *in vitro* experiments, *f_s_* simplifies to 1.5 by assuming that the lipid bilayer is completely insulative (i.e., σ_*m*_ ≈ 0), allowing one to easily estimate ΔΨ_*m*_ for a specific experiment or treatment. The dependence of the TMP on *θ* results in a potential gradient with a maximum near *θ* = 0 and minima at the poles.

Mathematical descriptions of pore formation following the induced TMP have also been reported. The Smoluchowski diffusion equation provided the original framework for pore formation.^44^ This partial differential equation describes the flux, *S*, of pores in and out of the membrane as a function of time and pore radius:

(5) $$S\left(t,r\right)=\frac{dN}{dt}-D\left(\frac{\partial N}{\partial r}+\frac{N}{k_{B}T}\frac{\partial U}{\partial r}\right),$$

where *N* is the pore density distribution function, *D* is the diffusion constant of the pores, *U* is the pore energy, *k_B_* is Boltzmann's constant, and *T* is the absolute temperature. In 1999, Neu and Krassowska derived an asymptotic reduction of Equation (5) to an easily solvable ordinary differential equation.^45^ Briefly, a quadratic term was introduced to describe the formation of hydrophilic pores beyond a critical radius. For small hydrophilic pores, a Bessel function is used to explain expansion. The intercept of these two curves represents a local energy minimum indicated by the sharp peak at *r* = 0.5 nm in Figure 1b. In summary, the pore energy can be described as:

(6) $$U\left(r\right)=\left\{\begin{array}{c} U_{*}{\left(\right.\frac{r}{r_{*}})}^{2}-\pi a_{p}r^{2}\Delta \Psi_{m}^{2},0\leq r\leq r_{*}\\ 2\pi r\gamma -\pi r^{2}\Gamma -\pi a_{p}r^{2}\Delta \Psi_{m}^{2}+{\left(\frac{C}{r}\right)}^{4},r_{*}\leq r\leq h \end{array}\right.,$$

where *U*_∗_ and *r*_∗_ are the critical energy and pore radius at the hydrophobic–hydrophilic transition, respectively; γ is the line tension per unit length of pore perimeter; and Γ is the surface tension per unit area of the intact membrane. The final quadratic term accounts for the steric repulsion between lipid heads lining the pore, where *C* is a constant chosen to match empirical data.^45–47^ The term 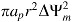 represents the capacitive contribution to the transition in which *a_p_* defines the dielectric permittivity of the porous membrane and is given as *ε*_0_(*κ*_*w*_ − *κ*_*m*_)∕2*h*. Here, *ε*_0_ is the dielectric permittivity of free space, and κ_*w*_ and κ_*m*_ are dielectric constants of water and the membrane, respectively. Figure 1b illustrates the behavior of this system for different values of ΔΨ_*m*_. Substituting Equation (6) into (5), rearranging, and simplifying with a number of assumptions, pore density *N*(*t*) is approximated by:

(7) $$\frac{dN}{dt}=\alpha e^{\beta^{2}}\left(1-\frac{N}{N_{0}}e^{-q\beta^{2}}\right),$$

in which *α* and *q* are fitting parameters, *β* is the ratio of ΔΨ_*m*_ to the electroporation threshold ΔΨ_*ep*_, and *N*_0_ is the pore density when ΔΨ_*m*_ = 0.^48^ A summary of the parameters presented in these equations can be found in Table 1. A significant body of work has shown agreement between these equations and experimental observations. Furthermore, researchers are now able to directly utilize these simplified equations via analytical or numerical methods for experiment planning, protocol optimization, and data analysis.

**Table 1.** Summary of Parameters Used in Basic Transmembrane Potential and Pore Formation Equations (Adapted from Sweeney^147^)

Symbol	Definition	Value or range	Units	Source
*R_c_*	Radius of a mammalian cell	5–50	μm	—
*k_B_*	Boltzmann constant	1.380649 × 10^−23^	J/K	—
*T*	Temperature	295	K	—
*σ*_*e*_	Extracellular fluid conductivity (saline)	1.6	S/m	—
*ε*_0_	Dielectric permittivity of vacuum	8.85 × 10^−12^	F/m	—
*h*	Lipid bilayer thickness	3–15	nm	DeBruin and Krassowska,^46^ Kotnik and Miklavcic^148^
*σ*_*i*_	Cytosol conductivity	0.1–1	S/m	DeBruin and Krassowska,^46^ Kotnik and Miklavcic^148^
*σ*_*m*_	Lipid bilayer conductivity	10^−10^ to 8.7 × 10^−6^	S/m	Gascoyne et al.,^149^ Hu et al.,^150^ Smith et al.^151^
*ε*_*m*_	Lipid bilayer dielectric permittivity	4.4 × 10^−11^	F/m	Smith et al.,^151^ Ye et al.^152^
*τ*_*m*_	Membrane charging constant	0.1–1	μs	Weaver and Chizmadzhev,^1^ Vasilkoski et al.^153^
*D*	Diffusion constant of pores	5 × 10^−14^	m^2^/s	Neu and Krassowska^45^
U_*_	Pore energy at transition	45	kT	Neu and Krassowska^45^
r_*_	Pore radius at transition	0.5–0.8	nm	Neu and Krassowska,^45^ Smith et al.^151^
*γ*	Normalized line tension of pore perimeter	1.8 × 10^−11^	J/m	Neu and Krassowska^45^
Γ	Normalized surface tension of membrane	10^−3^	J/m^2^	Neu and Krassowska^45^
*C*	Quadratic fitting parameter	9.67 × 10^−15^	J^1/4^m	Neu and Krassowska^45^
*a_p_*	Permittivity constant	6.9 × 10^−2^	F/m^2^	Neu and Krassowska^45^
*κ*_*w*_	Dielectric constant of water	80	—	Neu and Krassowska^45^
*κ*_*m*_	Dielectric constant of membrane	2	—	Neu and Krassowska^45^
*N*_0_	Equilibrium pore density	1.5 × 10^5^	cm^−2^	Debruin and Krassowska^46^
ΔΨ_*ep*_	Characteristic voltage of electroporation	258	mV	Debruin and Krassowska^46^
*α*	Fitting parameter	100	cm^−2^ms^−1^	Debruin and Krassowska^46^
*q*	Fitting parameter	2.46	—	Debruin and Krassowska^46^

### Electric field intensity, duration, and IRE

Permeability increase, pore formation, and induced biological effects depend on applied parameters, such as the amplitude, number, and length of pulses. ECT treatments typically use eight pulses with electric field magnitudes near 1 kV/cm.^49^ However, when a high number of pulses (60–100) of sufficient amplitude (0.5–1.0 kV/cm) are delivered, treated cells lose homeostatic equilibrium and die within minutes to hours.^50^ The exact mechanisms through which IRE causes cell death have not been fully elucidated, but a number of possible pathways such as direct electroconformational denaturation of macromolecules,^51,52^ induced depletion of adenosine triphosphate (ATP),^53^ local vascular disruptions,^49,54^ or electrolytic pH changes^55^ could contribute to cell injury.

Furthermore, it has been shown that Na^+^/K^+^ pumps play a major role in restoring contractility after electroporation of skeletal muscle, supporting the theory that chemical imbalances mediate eventual cell death.^56^ Ultimately, high amounts of ATP are required to restore disrupted chemical concentration gradients; depending on the number and lifespan of the pores, this ATP demand can outweigh what can be generated by the cell, leading to high levels of intracellular Ca^2+^ and eventual cell death.^57^ Notably, the temporal scale in which IRE lesions appear seems to vary depending on the tissue type, suggesting a moiety of death mechanisms.^58^ Future work evaluating these mechanisms more rigorously will be vital to our understanding of IRE-induced cell death.

The application of an electric field across a dielectric material—such as biological matter (cells/tissue)—results in resistive losses and subsequent generation of heat. Thus, supplying excessive electrical energy within a given time frame can cause thermal damage.^14,59,60^ This side effect can be alleviated by referencing the literature to use previously determined nonthermal parameters for specific tissues, by incorporating thermal mitigation strategies, or by modeling treatment beforehand to select pulse paradigms capable of nonthermal IRE.^61^

### Basics of tissue ablation with IRE

**FIG. 2.**
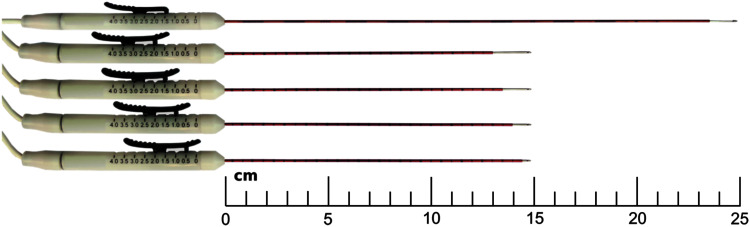
IRE is delivered through 25- or 15-cm long monopolar electrodes with varying degrees of exposure. IRE, irreversible electroporation.

IRE is typically performed as a minimally invasive percutaneous or laparoscopic procedure. Patients are sedated with general anesthesia, and neuromuscular blocking agents are prophylactically administered to mitigate muscle contractions that can occur due to electrical stimulation of nearby neurons. Although flat plate and endoscopic electrodes have been used, electrical energy is typically supplied through parallel needle electrodes 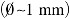 inserted directly into the tissue of interest (Fig. 2). Depending on the size of the lesion, IRE is performed with as little as two or up to six monopolar probes. Additionally, a single-insertion bipolar probe has been reported for certain small tumors in precarious locations. After insertion, probe locations can be verified via intraoperative imaging (ultrasound or computed tomography [CT]), and 50–100 pulses on the order of 100 μs in length are sequentially delivered between each electrode pair. IRE is synchronized with the absolute refractory period of the electrocardiogram (ECG) to mitigate the risk of electrical interference with cardiac myocytes and potential arrhythmia.^62^

The electric field distribution in the tissue drives treatment outcome—this is dictated by a variety of factors including electrode geometry/configuration, pulse parameters, and tissue properties. Important tissue-specific parameters pertaining to the production of nonthermal ablation are the dynamic electrical conductivity and lethal electric field threshold.^63^ Dynamic conductivity describes the evolution of the tissue's electrical conductivity when exposed to electric fields of increasing amplitude and can be determined through carefully planned experiments.^63,64^ The lethal threshold is a metric of the susceptibility of a certain tissue or cell type to IRE-induced cell death. It is dependent on the shape and amplitude of the characteristic waveform, number of pulses, and duration of the applied field, but for most tissues, this threshold is between 300 and 1000 V/cm when 100 pulses are applied.^65^

It is critical to note that the electric field threshold for a given tissue decreases as more pulses are applied but saturates after a certain number of pulses. Moreover, because minor Joule heating effects occur during treatment, increased pulse numbers result in local increases in conductivity at a rate of 1–3% per degree Celsius, which can also propagate the electric field and increase ablation volume. However, because IRE outcomes are heavily dependent on user inputs as well as characteristic properties of the tissue under study, the framework by which lethal thresholds are calculated must be taken into careful consideration. The minimum parameters that should be reported after IRE treatment are the number of pulses, pulse width (μs), frequency (Hz), and voltage-to-distance ratio (VDR, V/cm)—defined as the quotient of the applied potential (V) and electrode separation (cm). These allow one to gauge the intensity of treatment and provide a basic level of standardized reporting.

**FIG. 3.**
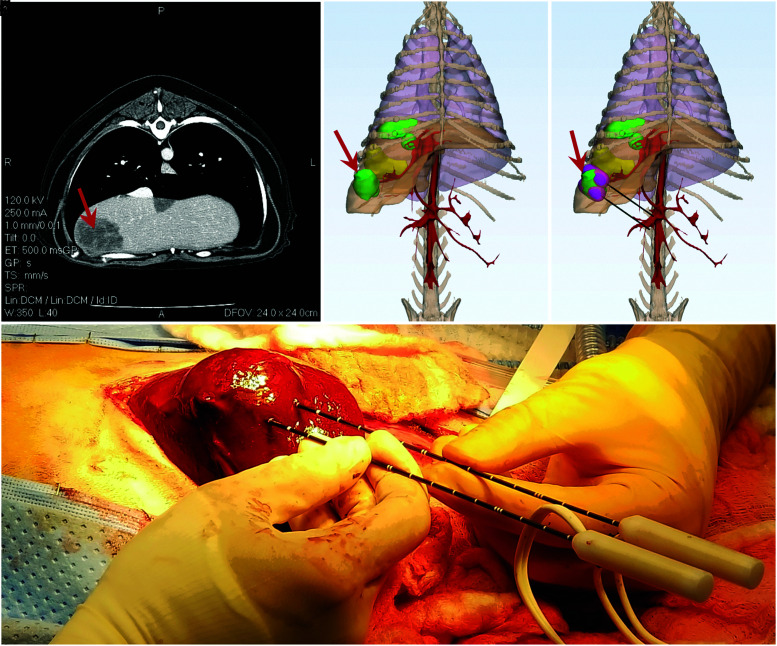
Pretreatment planning allows for prediction of ablation outcomes. For a canine case of multifocal liver cancer, this consists of **(a)** locating malignant tissue (red arrow) on diagnostic imaging, **(b)** reconstructing patient anatomy to assess tumor proximity to relevant structures, **(c)** identifying suitable electrode insertion pathway and estimating ablation volume (pink), and **(d)** using the pretreatment planning model to inform insertion tracts for optimal ablation outcomes.

Preclinical investigations of IRE often employ pretreatment planning tools to study the outcomes of certain pulse paradigms. Such models allow for the development of personal treatment plans for each patient, ensuring accurate probe placement, complete tumor coverage, and minimal temperature rise.^66,67^ A brief example of the treatment planning process can be seen in Figure 3. In the future, such planning modules will likely become a mainstay of human treatments, but no clinically accepted modeling procedure/algorithm has been introduced thus far.

## IRE as a Monotherapy

**FIG. 4.**
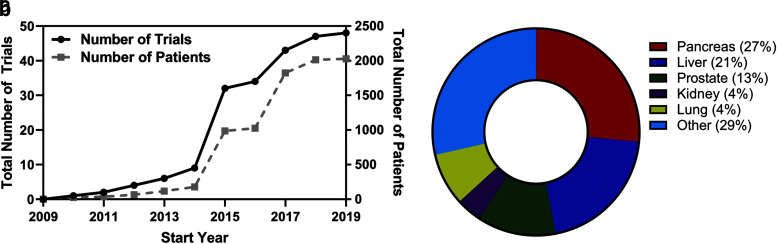
**(a)** Temporal trends in the cumulative number of interventional clinical trials investigating IRE and the corresponding number of patients associated with them. **(b)** Distribution of clinical trials based on cancer localization. Data acquired from ClinicalTrials.org on August 31, 2019.

Since its introduction in 2005, more than 50 clinical trials have been organized to study IRE (Fig. 4) and hundreds of articles demonstrating clinical outcomes have been published. IRE has helped more than 5500 patients with unresectable cancer, many of whom have participated in these trials. In subsequent sections, we review the major *in vivo* work and clinical studies that provide insight regarding the uses and outcomes of IRE. While we acknowledge many studies that have investigated IRE in the lung, kidney, brain, and other organ systems, we maintain a focus on the prostate, pancreas, and liver, as cancers in these locations pose specific opportunities that IRE can potentially address.

### Prostate

Prostate cancer (PCa) is the most common neoplasm in men, accounting for one in five new cancer cases.^68^ Although patients diagnosed with PCa have 5-year survival rates of nearly 100%, this disease is the second most deadly cancer in the male population.^69^ Available interventions including radical prostatectomy and radiotherapy tend to have negative consequences on sexual and urinary function^70^; thus, focal ablation has emerged as a viable alternative. IRE is particularly promising for patients with PCa due to fact that roughly 80% of these patients exhibit localized disease.^71^ Additionally, IRE may improve functional outcomes for PCa patients due to the presence of protein-rich structures near the prostate, including the neurovascular bundle, lower urinary sphincter, ejaculatory vesicles, and urethra—all of which are at risk when patients undergo resection or thermal ablation.

The first evaluation of IRE in the prostate was performed by Onik et al. in 2007.^22^ In this initial study, six healthy canine prostates were treated with IRE using VDRs up to 3000 V/cm. The urethra was spared even when purposefully ablated, and although necrosis was noted on directly ablated vessels, patency was maintained without evidence of thrombosis. In a follow-up study, Onik and Rubinsky evaluated early safety and efficacy of IRE in 16 human patients.^72^ At 3 weeks post-IRE, 93% of biopsies were negative and potency was preserved in all men potent before treatment. Subsequent reports sought to elaborate on these early findings. In 2013, Tsivian and Polascik performed bilateral ablations in the healthy prostate of 12 dogs and confirmed the ability of IRE to spare the urethra, rectum, and capsule when probes were placed in close vicinity (∼7 mm) to these structures.^73^ Additionally, no clinically significant side effects were observed and erectile function was maintained in all dogs.

**FIG. 5.**
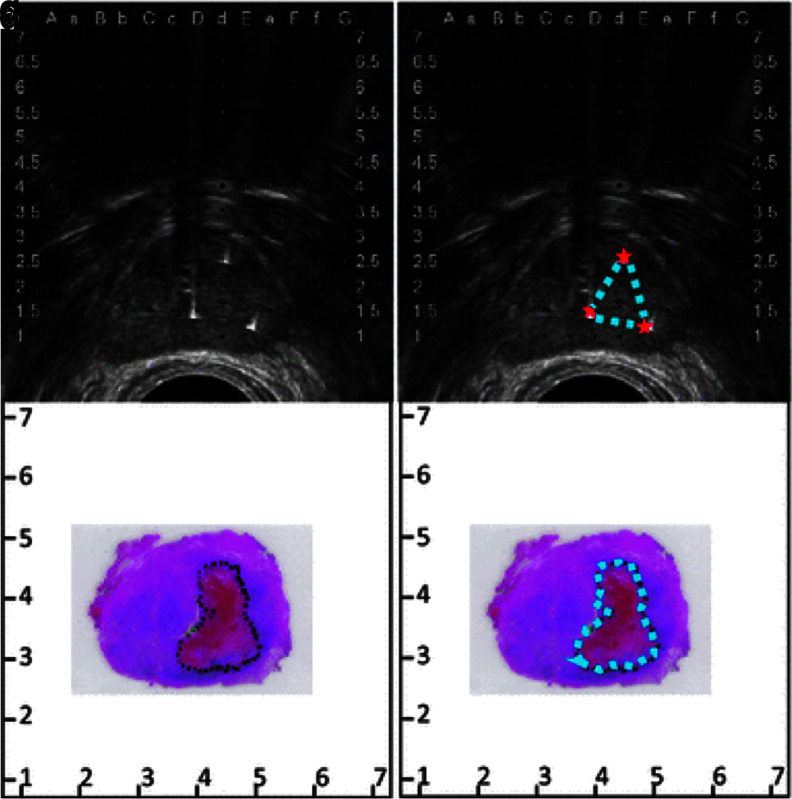
Ablation regions visualized on transrectal ultrasound correlate with those quantified histologically. **(a)** Perioperative ultrasound shows **(b)** the area circumscribed by the electrode configuration within the hypoechoic prostatic tissue. **(c)** Histology obtained after radical prostatectomy 4 weeks post-treatment shows **(d)** the area of ablation, which closely resembles the size, shape, and location of that visualized intraoperatively. Image originally found in van den Bos et al.^77^ reprinted under Creative Commons Attribution 4.0 International License.

The first report evaluating intermediate-term safety and feasibility in humans was published shortly thereafter.^74^ In this study, IRE was performed in 34 men with localized PCa. At a median follow-up of 6 months, potency and continence were preserved in 95% and 100% of men potent/continent before the study. From an oncological perspective, six patients had suspicious residual disease and one local failure was recorded. In 2015, a series of investigations evaluating quality of life (QoL),^75^ histopathological outcomes,^76^ and effects of electrode configuration on ablation outcomes^77^ were published on a 16-patient cohort that underwent IRE ablations followed by radical prostatectomy 4 weeks later. Here, it was shown that the mild adverse events resulting from IRE were mostly resolved by the 4-week time point. Slight declines were noted in urinary QoL while functional outcomes were retained. Histologically, sharp demarcations between ablated and viable tissue were noted, and they correlated well with the hypointense region seen on transrectal ultrasound (Fig. 5). Finally, it was found that a higher number of electrodes produced a larger ablation relative to the area circumscribed by the electrode configuration.^77^

A number of other clinical studies have since evaluated IRE outcomes for the ablation of PCa (Table 2). Recently, two larger patient cohorts have been assessed. In the first, safety, QoL, and short-term oncological outcomes were evaluated by van den Bos et al.^78^ Safety was again demonstrated while oncological control was 84% in-field and 76% whole-gland. The authors noted that after gaining experience with the procedure, margins were increased and NanoKnife^®^ operation became more efficient; oncological control with increased margins in the absence of system errors increased to 97% and 87%, respectively. The latter study by Guenther et al. was a retrospective analysis of longer term outcomes for 429 patients.^79^ This cohort was made up of patients with high-risk disease (73%), but 5-year recurrence rates were similar to that of radical prostatectomy at roughly 10%. Furthermore, IRE maintained urogenital function, including complete preservation of urinary continence, while only 3% of patients experienced erectile dysfunction 12 months after treatment. Although further randomized evaluations are necessary, these results support the safety and feasibility for treatment of patients with localized PCa, especially those with disease that is recurrent and/or not amenable to surgery/radiation.

**Table 2.** Summary of Clinical Irreversible Electroporation Studies of the Prostate

Source	IRE parameters					Patients/TRx	Clinical data							Comments
	Probe No., mean (range)	Separation, mm	VDR, V/cm	Pulses			Gleason grade				Recurrence, if/adj/oof	Adverse events, 1/2/3/4/5	Months,*^a^*[urinary continence; potency]^b^	
				No.	Length, μs		6	7	8	9				
Onik and Rubinsky^72^	4	10–15	1000–1500	90	70–100	16	7	6	3	0	1 (0/0/1)^c^	NR	6 [100%; 100%]	Adequate flow in NVB postoperative
Valerio et al.^74^	4 (2–6)	<20	NR	90	70	34	9	19	6	0	6^d^	12/10/0/0/0	6 [100%; 95%]	Average ablation volume of 12 mL
van den Bos et al.^75^	4 (2–6)	NR	1500 (1200–2100)^e^	90	90	16	0	8	8	0	N/A	15/8/1/0/0	1 [100%; NR]	Electrode configuration completely enveloped ablation, leaving no viable cells in 15 patients
Srimathveeravalli et al.^154^	4 (3–5)	10–15	1620 (1100–2080)^f^	70	90	6	5	0	0	1	3	NR	8 [100%; 100%]	700 V/cm electric field correlated with MRI
Ting et al.^155,g^	4.6 (3–6)	6–20	820–2880^f^	90	70	25	2	23	0	0	5 (0/4/1)	NR	6 [100%; NR]	Good oncological control achieved with low toxicity
Murray et al.^156^	4.8 (3–6)	10–20	2340 (1650–2700)^h^	90	NR	25	18	7	0	0	7 (4/NR/NR)	6/7/1/0/0	12 [92%; 96%]	Statistically insignificant increases in urinary and erectile function according to questionnaires
Scheltema et al.^157^	4.3 (3–6)	9–18	890–2910^f^	90	70 or 90	63/64	9	54	0	0	11 (7/0/4)	15/7/0/0/0	12 [100%; 77%]	Low safety margin (5 mm) and overcurrent correlated with risk of residual disease
Guenther et al.^80^	5 ± 1^g^	≤20	1518.13 ± 204.05^i^	NR	100	429/471	82	225	71	0	47	93/17/7/0/0	12 [100%; 97%]	Comparable 5-year RFS to RPE with improved urogenital outcomes

Clinical studies with 5+ patients were included.

^a^ Here, months are either the median follow-up or the latest time point at which all patients were evaluated.

^b^ Percentage calculated from patients potent before treatment.

^c^ One patient refused postoperative biopsy.

^d^ Only one in-field recurrence was verified by biopsy.

^e^ Target, range.

^f^ Estimated from stated voltage/separation.

^g^ Two patients had transurethral resection (TURP) of the prostate during procedure.

^h^ Median (interquartile range).

^i^ Mean ± standard deviation.

adj, adjacent; if, in-field; IRE, irreversible electroporation; MRI, magnetic resonance imaging; NR, not reported; NVB, neurovascular bundle; oof, out-of-field; RPE, radical prostatectomy; RFS, recurrence-free survival; TRx, treatments; VDR, voltage-to-distance ratio.

### Pancreas

**FIG. 6.**
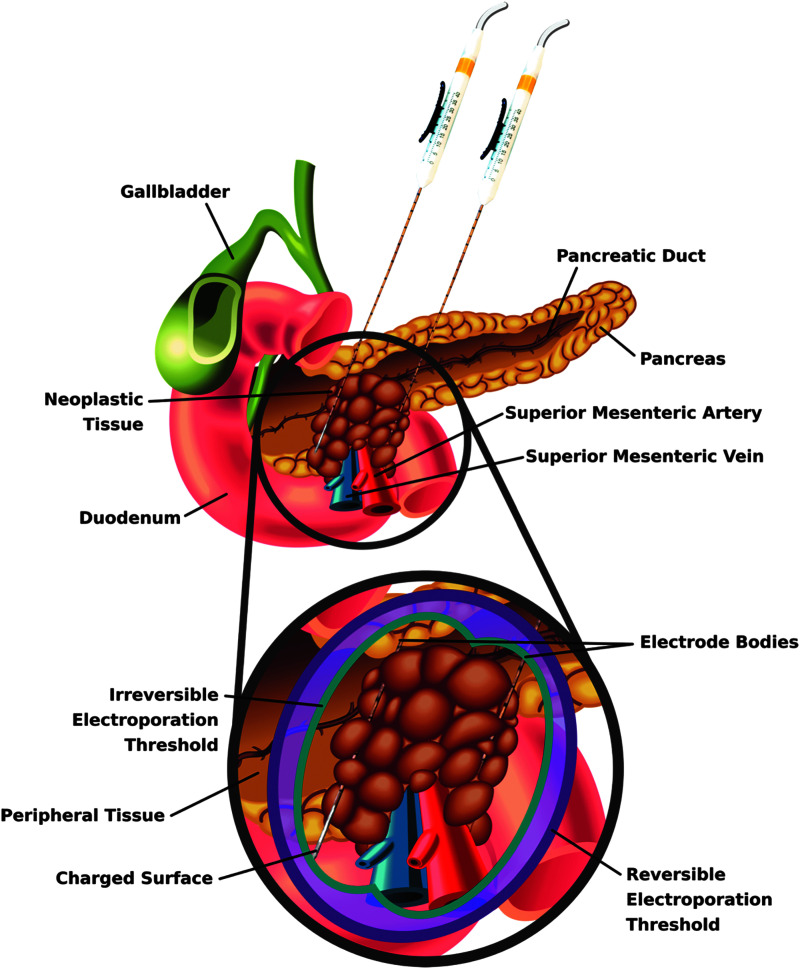
Schematic depicting generalized electrode placement and resulting treatment zone for a pancreatic tumor encasing the superior mesenteric vessels. The proximity of the pancreas to these vessels and other vasculature limits interventional options for a large number of patients. In such cases, IRE has shown promise as it allows for focal ablation of the tumor without long-term injury to these critical proteinaceous structures. Additionally, the zone of reversible electroporation could be used in the future to increase uptake of adjuvant molecules and/or chemotherapeutics in the periphery of the ablation, further increasing efficacy.

So far, unresectable pancreatic tumors present perhaps the largest window of opportunity for IRE as a technique capable of drastically improving clinical outcomes. Patients with pancreatic cancer have extremely low 5-year survival (9%),^68^ and less than 20% are candidates for surgery.^80^ The pancreas is situated near the celiac trunk, hepatic artery, and superior mesenteric vessels, contributing to its surgical inaccessibility (Fig. 6). Thermal ablation is associated with high morbidity when applied to pancreatic tumors due to the presence of these fragile structures.^81^ Thus, IRE is an emerging tool that could play a critical role in the future management of patients with locally advanced disease.

To date, roughly 30 articles have evaluated IRE *in vivo* for focal ablation of pancreatic tissue. The first of these was performed by Charpentier et al. in swine.^82^ The authors of this study used two monopolar needle electrodes with an induced voltage of 1500 V and spacing of 10–15 mm to ablate healthy pancreas. All pigs tolerated the procedure without complication and pancreatic ducts were preserved. Bower et al. published a similar study shortly thereafter, confirming the preservation of vascular structures and reporting the feasibility of both monopolar and bipolar IRE probes for pancreatic ablations.^23^ The first clinical study of pancreatic IRE was performed by Martin et al. in 2012.^83^ Here, 27 patients with locally advanced pancreatic adenocarcinoma (LAPC) were treated with IRE at a target VDR of 1500 V/cm. One hundred percent ablation success was achieved with only four potential IRE-related adverse events, and no evidence of recurrence was observed within the 90-day follow-up period. In a similar study, Narayanan et al. performed the first percutaneous IRE treatments in a cohort of patients with unresectable or metastatic disease under CT guidance.^84^ Of the 15 patients, 2 were downstaged and underwent margin-negative resection. In the remaining 13 patients, 46% had stable disease at last follow-up. In 2015, Martin et al. published a study evaluating IRE in 150 patients with LAPC.^85^ Patients receiving treatment with IRE had an overall survival of 18 months and local progression-free survival of 10.7 months. Including another 50 patients in whom IRE was used for margin accentuation, overall survival for the 200-patient cohort was 24.9 months.

In recent years, IRE has been associated with overall survival of up to 27 months for patients with unresectable LAPC,^86^ and response rates commonly exceed 70%.^87–89^ In addition to potential survival improvements, some patients have been able to lower narcotic intake^83^ after treatment and others have shown major improvements in QoL.^89^ Depending on the extent of vascular involvement, downstaging occurs in 5–10% of cases, allowing for follow-up resection.^86,90,91^ Despite the promising results reported in some studies, a limited number of institutions have adopted IRE (Table 3). This could be due to inherent difficulties in treating advanced pancreatic tumors, discrepancies in patient selection, and differences in institutional protocols for IRE application. Moreover, postoperative imaging has proven more difficult for assessing ablation outcomes for pancreatic lesions, which could contribute to these inconsistent findings.^93^ Although many studies have reported prolonged survival after IRE, future randomized studies are critically needed to directly compare the safety and efficacy of IRE against alternative treatments.

**Table 3.** Summary of Clinical Irreversible Electroporation Studies of the Pancreas

Source	Method	IRE parameters			Patients/TRx	Clinical data					Outcomes/comments
		Electrodes		Sep, mean or range, mm		Tumor size, median (range), cm	Months,*^a^*recurrence (local/distant)	Adverse events (1/2/3/4/5)^b^	Median OS, after IRE, mo	Months,*^c^*[CR/PR/SD/PD %]^d^	
		Type	Number, mean (range)								
Martin et al.^84^	Op (26), P (1)	M (23), B (4)	4^e^ (3–5)	NR	27/27	3 (1–5.5)	3 (1/NR)	18	NR	3 [96.2/NR/NR/NR]	Reduced narcotic use and 4 possible IRE-related complications
Narayanan et al.^85^	P	M B	NR	≤22	14/15	3.3 (2.5–7)	4 (3/3)	NR	NR	4 [14/0/43/43]^f^	Patients with metastatic disease had no survival benefit
Martin et al.^88^	Op (52), L (2)	M (48), B (6)	4^e^ (1–6)	NR	54/54	3.2 (1–5.5)	15 (15/12)	67	20.2	3 [85/NR/NR/NR]	OS with IRE+chemo of 20.2 months versus 11 for chemo alone
Mansson et al.^158^	P	M	NR	≤20	5/5	3.5 (1–4)	6 (0/2)	0 grade 3+	NR	6 [40/NR/NR/20]	Disease downstaged in 1 patient followed by R0 resection
Paiella et al.^159^	Op	M	2–6	10–20	10/10	3	7.6 (3/3)	9/4	7.5	7.6 [0/40/30/30]	Stage III patients that were unresponsive to chemotherapy
Martin et al.^86^	Op	M	4 (2–6)	NR	150/150	3 (1.6–7)	29 (6/NR)	100 (36/32/22/8/2)^g^	18	3 [95/0/0/5]	LPFS of 10.7 months IRE shows nearly 2 × survival versus standard of care
Kluger et al.^160^	Op	M	4 (3–6)	NR	24/29	2.7 (2.4–4)^h^	8.69 (11%/47%)	10 grade 3+	7.71	NR	Treated T4 tumors encasing the celiac artery or SMA
Månsson et al.^91^	P	M	5.58 (3–6)	NR	24/24	3.5 (1.5–4.5)	6.1 (14/13)	11 (8/3)^i^	7	6.1 [42/0/0/58]	Downstaging in 2 patients. No recurrence trends based on tumor size
Lambert et al.^161^	Op (19), P (2)	M	2	15–20	21/21	3.82 ± 1.2^j^	NR	7	10.2	NR	Matched cohort showed survival of 9.3 months (*p* = 0.053)
Yan et al.^162^	Op	M	4^e^ (2–6)	20	25/25	4.2 (2.8–4.9)	NR	4	NR	[0/36/28/36]	Treated large T3 tumors with 13 total complications, 92% of which were ≤grade 3
Belfiore et al.^90^	P	M	3.6 (2–5)	NR	29/29	94 (39–170)^k^	29 (3%)	0 major^i^	14	6 [0/100/0/0]	Downstaging in 3 patients. Mean Karnofsky score improved by 28 points at 3 months
Narayanan et al.^163^	P	M (45), B (5)	3.4 (1–6)	18–22	50/50	3.2 ± 1.3^j^ (1.5–8)	NR	45 (35/10)^l^	14.2	NR	Downstaging in 3 patients. Tumor size (≤3 cm) only significant predictor of OS
Scheffer et al.^164^	P	M	6^e^ (3–9)	15–24	25/25	4 (3.3–5)	NR	23 (12/11)^l^	11	NR	Median EFS from IRE of 8 months. Only 3 patients had signs of pancreatitis
Vogel et al.^165^	Op	M	3–6	≤25	15/15	NR	NR	53%	16^m^	NR	IRE performed after induction chemotherapy
Zhang et al.^166^	P	M	NR	15–20	15/15	3.5 (2–6.7)	NR	NR	NR	1 [2/9/3/1]	Mild complications were relieved within 24 h. No rise in amylase was observed
Leen et al.^87^	P	M	2	20	75/75	3.47 ± 1.2^j^	11.7 (2/26)	25 (12/6/1/0/6)	27	3 [0/31/66/3]	Downstaging in 4 patients. Median PFS was 15 months
Sugimoto et al.^167^	Op (4), P (4)	M	4 (3–4)	≤24	8/8	2.95 (2.6–4.25)^h^	17.5 (3/5)	9 (5/4)^l^	17.5	NR	LPFS of 12 months after IRE. 63% of patients developed distant metastases
Flak et al.^92^	Op (1), P (32)	M	5^e^ (3–9)	15–20	33/40	3.0 (1.8–5.2)	NR	NR	10.7	NR	Downstaging of 3 patients. OS = 16.7 months for tumors ≤3.5 cm
Liu et al.^168^	Op (16), P (38)	M	2	16 ± 4 (10–20)	54/54	4.97 ± 1.64^j,k^	NR	54 (50/4)^n^	16.2/20.3 (IRE/IRE+Chemo)^o^	NR	Chemotherapy+IRE more beneficial for patients with stage IV disease
Månsson et al.^169^	P	M	4–6	≤20	24/24	3.5 (3–4)^h^	5.8 (8/11)	18 (12/6)^n^	11.5	0 [96/NR/NR/NR]	Percutaneous IRE used as first-line therapy

Articles reporting IRE in 5+ patients were included.

^a^ Here, months are typically the median follow-up. In some cases where median follow-up is not reported, this value is the latest time point at which recurrence is assessed.

^b^ According to the CTCAE v4.

^c^ Here, months are typically the latest time point at which local progression was assessed, but in some cases where this is not reported, the median follow-up is used. Time points listed as “0” are immediate assessments within 1–2 days of treatment.

^d^ Evaluated according to the RECIST.

^e^ Median.

^f^ Two patients were downstaged and underwent resection at 4 and 5 months, respectively.

^g^ Graded according to a scale introduced by Martin et al.^92^

^h^ Median (interquartile range).

^i^ Major/minor complications according to the Society of Interventional Radiology guidelines.

^j^ Mean ± standard deviation.

^k^ Volume, mL.

^l^ Major/minor according to the CTCAE v4.

^m^ Major/minor according to the Clavien–Dindo criteria.

^n^ From diagnosis, for patients with stage III disease. Stage IV OS was 11.6 and 15.6 (*p* = 0.04), respectively.

^o^ From diagnosis.

B, bipolar; CR, complete response; CTCAE, Common Terminology Criteria for Adverse Events; EFS, event-free survival; L, laparoscopic; LPFS, local progression-free survival; M, monopolar; Op, open; OS, overall survival; P, percutaneous; PD, progressive disease; PFS, progression-free survival; PR, partial response; RECIST, Response Evaluation Criteria In Solid Tumors; SD, stable disease.

### Liver

Hepatocellular carcinoma (HCC) represents the vast majority of liver malignancies and is the third most common cause of cancer-related mortality.^94^ When detected early, curative treatment for HCC can be achieved with resection or transplantation, but less than 15% of patients fall into this category.^95^ The liver is also frequently a site of metastasis, especially for primary tumors of the gastrointestinal tract. Focal thermal ablation has become a mainstay in the management of liver lesions and has shown similar oncological outcomes to resection with limited complications and without reducing transplant exception points.^96,97^ Despite this, thermal ablation is often not an option due to the presence of tumors on or near hepatic blood vessels or biliary structures, and patients with underlying liver dysfunction have increased rates of post-treatment abscess formation after thermal ablation.^98^ The nonthermal nature of IRE allows it to overcome many of these limitations, and its role in the management of hepatic masses is still being fully established.

The first *in vivo* study of IRE for liver ablation used flat plate electrodes and a single, 20 ms monopolar pulse with 1 kV/cm amplitude to create reproducible regions of “endothelial necrosis, thrombus formation, vascular compromise, and vacuolar degeneration” within 3 h of treatment in rats.^18^ Shortly after this publication, needle electrodes were used to generate ablations in a large animal model,^20^ and the ability to perform percutaneous IRE in the liver and observe ablation in real time via ultrasound was demonstrated.^19^

These early findings were first translated to the clinic when Thomson et al. found IRE to have an acceptable safety profile for the ablation of the liver, kidney, and lung tumors, including metastases from primary tumors elsewhere in the body.^99^ In several subsequent studies, IRE was shown to be suitable for ablating tumors near vital hepatic structures.^100–104^ The ability to treat these tumors has opened the door to many clinical investigations (Table 4). Some of these reports have investigated functional deficits following IRE and have found that biomarkers of hepatic function tend to rise transiently (1–2 days) but return to baseline within a few days, verifying safety.^103,105^ Notably, Bhutiani et al. found that IRE had a similar 6-month success rate but was more tolerable than microwave ablation for patients with compromised liver function (Child-Pugh B).^106^ IRE-treated patients also had shorter hospital stays and lower rates of re-admission, likely due to lower indiscriminate effects on hepatic tissue.

**Table 4.** Summary of Clinical Irreversible Electroporation Studies of the Liver

Source	Method	IRE parameters				Patients/TRx	Clinical data					Comments
		Electrodes		Sep, mm	VDR, V/cm or V, median (range)		Tumor type	Tumor size, median (range), cm	Months,*^a^*recurrence (local/distant)	Complications (major/minor)^p^	Months*^b^*[CR/SD/PD %]^c^	
		Type	Number									
Thomson et al.^99^	P	M, B^d^	1–5	≤25	1500–3000 V	25/63	HCC (17), CRLM (15), BCLM (13), other (17)	2.4 (1–8.8)	NR	NC	3; HCC [83/0/17], CRLM [47/0/53], BCLM [77/0/23], O [12/66/23]	No tumor control for metastases >5 cm. Complete ablation in 15/18 primary HCC tumors
Kingham et al.^100^	Op (22), P (6)	M	2.6 (2–5)	≤25	1781 V(1303–3000)	28/65	HCC (2), CRLM (21), O (5)	1.0 (0.5–5)	6 (3/NR)	NC	NR^e^	Tumors treated close to vessels purposefully with low adverse events and one occlusion
Cannon et al.^101^	Op (2), P (28), L (14)	M	3 (2–5)	5–20	3000 V	44/48	HCC (14), CRLM (20), BCLM (2), O (8)	1.1–11	12 (18/NR)	9/5	0 [100/0/0]	3, 6, and 12 months LRFS = 100%, 100%, and 98% for lesions <3 cm. Increased recurrence for larger tumors
Cheung et al.^170^	P	M, B	NR	NR	≥1000 V/cm	11/18	HCC	2.44^f^ (1–6.1)	18 (11/5)	11	14 [72/NR/28]	CR in 93% of lesions ≤3 cm
Scheffer et al.^171^	Op	M	3 (2–5)	20 ± 2	1500 V/cm	10/10	CRLM	2.4	N/A	1	N/A^g^	TTC staining corresponded with IOUS and showed vitality of tumor in 8/9 specimens
Eisele et al.^172^	Op (2), P (7), L (4)	M	4.2 (4–5)	NR	NR	13/14	HCC (5), CRLM (6), O (2)	1.45 (0.6–2.4)	6 (13)	NR	NR^h^	Recurrences occurred only for large (>2 cm) or multifocal tumors
Hosein et al.^173^	P	M (30), B (6)	NR	11–24	NR	29/58	CRLM	2.7 (1.2–7)	10.7 (4/15)	NC^i^	10.7 [18/46/18]	Treated unresectable lesions w/contraindications for TA. Median PFS of 4 months
Silk et al.^103^	P	M	3 (2–6)^j^	16 (8–30)^j^	1510 V/cm (904–1982)^j^	11/22	CRLM (16), O (6)	3.0 (1–4.7)	9 (6/NR)	NR	NR	Increased bilirubin/alkaline phosphatase in 4 patients
Dollinger et al.^174^	P	M	4 (2–6)^j^	NR	1650–3000 V	56/114	HCC (45), CRLM (44), BCLM (6), O (19)	2.2 (0.2–6.3)	NR	19 (12/7)^k^	NR	Major complication rate of 7.1% in 85 procedures
Niessen et al.^175^	P	M	3.4 (2–6)	11.9 (6–29)	1000–3000 V	25/48	HCC (22), CRLM (16), O (10)	1.7^f^ (0.7–2.6)	5 (14/NR)	NR	NR	Metastatic and large tumors (>5 cm^3^) showed increased recurrence
Eller et al.^176^	P	M	2.8 (2–4)	≤20	1500 V/cm	14/18	HCC (4), CRLM (8), O (2)	1.8 (0.6–2.6)	13 (4/5)	4/NR^l^	NR	Only 2 of 12 successfully treated lesions recurred locally at 388 days
Niessen et al.^177^	P	M	3.1 (2–6)	10–20	1500 V/cm	34/65	HCC (33), CRLM (22), O (10)	2.4 (0.2–7.1)	6 (9/NR)	14 (6/8)	12 [61.9/NR/NR]	Complete initial ablation in 62/65 tumors. 12 months LRFS = 75%
Bhutiani et al.^106^	P (10), L (20)	M	NR	15–24	NR	30/30	HCC	3.0 (2–3.3)	6 (1/NR)	8	6 [97/0/3]	Patients with compromised liver function tolerated IRE better than MWA
Granata et al.^178^	P	M	4 (3–5)	NR	NR	20/24	HCC	2.0^f^ (1–3)	3 (2/NR)	2 (0/2)	6 [91.7/NR/NR]	MRI, CT, and CEUS show similar accuracy for evaluating IRE lesions
Frühling et al.^179^	P	M	3–6	≤ 20	NR	30/38	HCC (8), CRLM (23), O (7)	2.4 (0.8–4.0)	6 (12/NR)	7 (1/6)	6 [30/NR/NR]	High local control with minimal side effects
Langan et al.^180^	Op	M	NR	≤ 25	NR	40/77	HCC (7), CRLM (57), O (13)	1.3 (0.5–6)	25.7 (10/NR)	14^m^	25.7 [87/NR/NR]	Tumor size and BMI associated with risk of recurrence
Niessen et al.^181^	P	M	2–6	7–20	1650–3000 V	71/103	HCC (43), CRLM (42), O (18)	1.9 (0.4–4.5)	35.7 (33/NR)	12 (5/7)	1.5 [92.2/NR/NR]	Median OS of 26.3 months shortened to 9.5 months for tumors >3 cm
Barabasch et al.^182^	P	M	2–5	15 (10–20)	≤3000 V	27/37	CRLM (15), BCLM (4), O (8)	6.4 ± 11.39^n^	NR	NR	0 [91.9/NR/NR]	Average ablation volume of 39.8 mL decreased continuously to 5.1 mL at 8 weeks
Mafeld et al.^108^	P	M	3 (2–7)	10–20	1500 V/cm	52/59	HCC (20), CRLM (28), BCLM (1), O (7)	2.4^f^ (0.7–5.2)	12 (23/NR)	9^o^	1.5 [75/NR/NR]	44% PFS at 12 months. Median OS of 38 months. Higher survival for HCC and small lesions (<2 cm)

Articles reporting IRE in 5+ patients were included.

^a^ Here, months are typically the median follow-up. In some cases where median follow-up is not reported, this value is the latest time point at which recurrence is assessed.

^b^ Here, months are typically the latest time point at which local progression was assessed, but in some cases where this is not reported, the median follow-up is used. Time points listed as “0” are immediate assessments within 1–2 days of treatment.

^c^ Determined by the RECIST.

^d^ Bipolar probe used for lesions that were small (<2.5 cm) or difficult to access.

^e^ Initial response rate was 98.1% and combined local failure rate was 7.5%.

^f^ Mean.

^g^ Ninety percent of treatments were technically successful (delivered all pulses+complete tumor coverage).

^h^ Three of 14 tumors were incompletely ablated, all after percutaneous IRE.

^i^ Two patients had transient cardiac arrhythmias, one had transient ventricular arrhythmia, and one had atrial fibrillation.

^j^ Median, range.

^k^ Of the seven major complications that occurred, four were abscesses and were associated with bilioenteric anastomosis (3/4).

^l^ Only major complications were reported.

^m^ There were 4 IRE-specific complications and 1 complication of the 14 patients who only received IRE.

^n^ Volume measured in milliliters (mean ± standard deviation).

^o^ Evaluated according to the CIRSE criteria. Grade 1: *n* = 5; Grade 3: *n* = 1; Grade 4: *n* = 2; Grade 6: *n* = 1.

^p^ According to the Society for Interventional Radiology.

BCLM, breast cancer liver metastasis; CEUS, contrast enhanced ultrasound; CIRSE, cardiovascular and interventional radiological society of Europe; CRLM, colorectal liver metastasis; CT, computed tomography; HCC, hepatocellular carcinoma; IOUS, intraoperative ultrasound; LRFS, local recurrence-free survival; MWA, microwave ablation; NC, not categorized; O, other.

In recent years, reports with intermediate to long-term follow-up have shown that IRE produces acceptable oncological outcomes for patients with unresectable disease, especially for small tumors.^107,108^ For tumors larger than 2–4 cm, overall and recurrence-free survival tend to decline, but modifications to the treatment paradigm in the future may allow for higher efficacy in larger lesions.^109^ Besides large tumors, the only other major contraindication for performing hepatic IRE is the presence of metallic implants in close proximity to the tumor, which has been shown to adversely affect progression-free survival.^110^ Varying degrees of complication have been observed with IRE in liver tumors,^100,103,108^ likely due to the learning curve inherent in the adoption of this new treatment modality.^111^ In recent retrospective analyses, however, the overall complication rate of hepatic IRE was similar to thermal ablation despite being used as a salvage therapy in many cases of advanced disease.^112,113^ In summary, IRE appears to be a promising option for precarious, central hepatic tumors abutting proteinaceous structures such as the biliary tree or portal vein, but again, randomized studies are needed to further delineate its oncological outcomes in comparison to existing focal therapies.

## Alternative Applications and Future Directions

### Cardiovascular

IRE may be a useful addition to the technological repertoire for cardiac ablation and other vascular applications where focal destruction of aberrant cells is desired, namely in conditions such as atrial fibrillation, restenosis, and resistant hypertension. Although it was first noted that intense electric fields arising during defibrillation may create “sarcolemmal microlesions” in 1987,^114^ IRE was not intentionally pursued for clinical cardiovascular applications until the pioneering work of Maor and Rubinsky appeared in the late 2000s.^15,115,116^

Early investigations applied IRE to rat carotid arteries and demonstrated ablation of vascular smooth muscle cells (VSMCs) without complication and without macroscopic damage to acellular vascular architecture^15^ using a wide range of pulse parameters.^117^ Later reports showed that VSMCs are eliminated over the course of 72 h without injury to elastic lamella, collagen fibers, or proteoglycans, and endothelial cells regenerated by 7 days.^118^ IRE has also been shown to mitigate restenosis following angioplasty,^119^ and custom devices capable of endovascular pulse delivery have been developed.^120^ Although not covered in depth here, these findings led to a series of articles investigating the use of IRE as a tissue decellularization technique, which was initially demonstrated both *in situ* and *ex vivo*.^118,121^

Lavee et al. were the first to use IRE to correct arrhythmogenic regions of the heart. Schemes using between 8 and 32 pulses generated completely transmural, epicardial atrial ablations in swine.^122^ Circular ablation catheters were introduced by Wittkampf et al. and were shown to safely generate clinically relevant lesions in pulmonary vein (PV) ostia^123^ and venricles.^124^ Van Driel et al. demonstrated a reduction in PV stenosis after catheter-based IRE versus radiofrequency ablation,^125^ and a later study demonstrated similar PV ablations with a commercially available catheter in canines.^126^ Other relevant work has shown that IRE can be used to ablate Purkinje fibers *ex vivo* and reduce vulnerability to ventricular fibrillation.^127^ Finally, one human study has been performed in which IRE was used successfully to electrically isolate PVs in 22 patients.^128^ Ablation times were less than 1 min, and 1-month follow-up visits indicated uneventful recovery. These emerging data support the further exploration of IRE for cardiovascular applications.

### Immune modulation and synergy with immunotherapy

Recently, the ability of focal tumor therapies to elicit systemic immune activation has garnered much attention. Immune modulation in response to IRE treatment has been investigated in a number of *in vitro* and *in vivo* studies. The first of these showed a decline in immune cell populations—CD4^+^ and CD8^+^ T lymphocytes, antigen-presenting cells, macrophages, and natural killer (NK) cells—over the course of 6 h after IRE treatment, indicating the ability of IRE to form substantial ablations without relying on antitumor immunity.^129^ A more comprehensive study evaluating immune cell populations and cytokines produced up to 21 days after treatment in an immune-competent rat osteosarcoma model found significantly increased CD3^+^ and CD4^+^ T lymphocytes, a higher CD4^+^/CD8^+^ ratio, and modified cytokine expression in IRE versus sham and surgery control groups.^130^

It was later shown that immunocompetent mice have a better local response to IRE and improved survival versus their immunodeficient counterparts.^131^ Furthermore, recent *in vitro* work has shown that melanoma cells ablated with IRE release substantially more protein and TRP-2 antigen than those treated with thermal ablation (heating and cryoablation).^132^ Additional analyses showed that protein released from IRE-treated cells was more efficient at inducing T cell proliferation than that released from heat- or cryotherapy-treated cells, supporting the observation that IRE reduces the rate of metastases in a rabbit VX2 model^133^ and exhibits a stronger abscopal effect than thermal ablation.^134^ Improved immune response versus other modalities could be due to release of intracellular contents or direct modulation of cell signaling, but further investigation is warranted.^135^

To assess the translational potential of these early findings, Lin et al. used IRE alone or alongside allogenic NK cell therapy in 71 patients with stage III or IV pancreatic cancer.^136^ IRE with NK therapy increased survival of patients with either stage III or IV disease and produced substantial increases in CD4^+^ and CD8^+^ T lymphocytes, NK cells, and B cells for stage III patients. Interestingly, Th2 cytokine (IL-4, IL-10) levels remained relatively unchanged while Th1 cytokines (IL-2 and IFN-γ) increased in response to both treatments, consistent with preclinical findings.^130^ This initial study, along with a similar investigation for patients with unresectable liver cancer, indicates that IRE could play a “priming” role by preparing the tumor microenvironment for effective exploitation by immunotherapy.^137^

Further clinical examination has shown that immune modulation occurs quickly after IRE and that attenuation of the highly immunosuppressive environment, as seen by a reduction in regulatory T cells, can be achieved for at least 2 weeks.^138,139^ These results are supported by the finding that IRE and anti-PD1 immunotherapy work synergistically to improve survival in a murine model of pancreatic cancer.^140^ This study again showed the presence of immune memory that was able to address tumor rechallenge. Similar to the findings of He et al.,^138^ infiltrating CD8^+^ T lymphocytes were associated with improved survival. These results are paving the way toward the clinical translation of combinatorial treatment strategies that capitalize on the ability of IRE to create an immunostimulatory microenvironment.

### High-frequency IRE

**FIG. 7.**
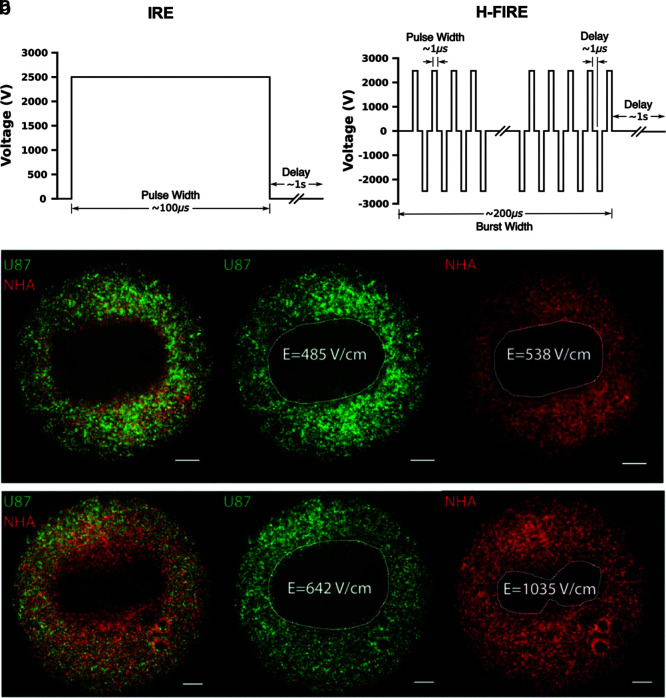
H-FIRE selectively targets malignant cells. IRE and H-FIRE employ voltage waveforms **(a)** with different characteristic frequencies, which result in unique biological effects. IRE-treated malignant (U251) and healthy (NHA) astrocytes **(b,** top**)** exhibit similar electric field thresholds for cell death. However, lethal electric field thresholds for H-FIRE **(b,** bottom**)** are much lower for U251 cells than NHAs, demonstrating the capacity of H-FIRE to target malignant phenotypes. **(b)** Published in Ivey et al.^144^ reprinted under Creative Commons Attribution 4.0 International License. H-FIRE, high-frequency irreversible electroporation. Scale bar represents 1 mm.

In 2011, Arena et al. numerically showed that short pulses (500 ns–2 μs) of alternating polarity may be more favorable for predictable tissue ablation in heterogeneous tissues than long monopolar pulses.^141^ In this initial computational analysis, a tissue domain consisting of an external layer of skin surrounding a cylinder of fat was modeled to study the extent of electroporation induced by pulsatile voltage waveforms with different characteristic frequencies between 250 kHz and 2 MHz. High-frequency voltage waveforms were shown to more uniformly penetrate the heterogeneous system. A follow-up study introduced the term high-frequency irreversible electroporation (H-FIRE, Fig. 7a) and showed the ability of these waveforms to generate nonthermal ablations in rat brain without muscle contractions.^142^ H-FIRE has since been used to treat intracranial malignancies in canines, and low-amplitude H-FIRE waveforms have been used to transiently disrupt the blood–brain barrier.^143^

In addition to the aforementioned advantages, *in vitro* work has shown that H-FIRE may exhibit selectivity toward malignant phenotypes (Fig. 7b).^144^ Furthermore, H-FIRE ablation in a 4T1 murine mammary tumor model stimulated a local inflammatory response and resulted in systemic immune activation capable of reducing distant metastases, suggesting that this technology is well suited for both standalone and combinatorial treatment strategies.^145^ In its first clinical evaluation, H-FIRE was used to treat PCa in 40 men without ECG synchronization.^146^ Four weeks after treatment, evaluated patients had complete preservation of urinary (40/40) and sexual (14/14) function. Additionally, no cardiac-related side effects were noted. These emerging data support the notion that H-FIRE will likely augment the clinical efficacy of high-voltage PEFs in the coming years. Future studies investigating oncological outcomes in larger randomized patient cohorts are warranted and will be critical to this development.

## Concluding Remarks

IRE has an array of potential advantages over existing technologies. Although promising results have been demonstrated, dissemination of knowledge and training is critical to the widespread adoption of IRE. Differences in physician experience, patient inclusion criteria, and reporting have led to inconsistencies in the application of IRE. Additionally, studies thus far have primarily evaluated IRE in patients with advanced disease and with numerous comorbidities. Despite this, IRE has shown to be a viable option for certain patients diagnosed with tumors of the prostate, liver, kidney, and pancreas. With proper patient selection and choice of pulse paradigms, IRE can improve outcomes as demonstrated by the *in vivo* and clinical results provided herein. Future developments involving combinatorial therapeutic regimens and alternate waveforms have shown exciting early results, and further evaluation will likely expand the clinical impact of IRE in the coming years.
